# Pathological complete response and residual DCIS following neoadjuvant chemotherapy for breast carcinoma

**DOI:** 10.1038/sj.bjc.6602950

**Published:** 2006-01-17

**Authors:** R L Jones, S R Lakhani, A E Ring, S Ashley, G Walsh, I E Smith

**Affiliations:** 1 Breast Unit, Royal Marsden NHS Trust, Fulham Road, London SW3 6JJ, UK; 2 Molecular and Cellular Pathology, School of Medicine, University of Queensland, Mayne Medical School, Herston Road, Herston, QLD 4006, Australia

**Keywords:** breast cancer, neoadjuvant chemotherapy, pathological complete response, residual ductal carcinoma *in situ* (DCIS), residual invasive carcinoma, prognosis

## Abstract

Patients who have no residual invasive cancer following neoadjuvant chemotherapy for breast carcinoma have a better overall survival than those with residual disease. Many classification systems assessing pathological response to neoadjuvant chemotherapy include residual ductal carcinoma *in situ* (DCIS) only in the definition of pathological complete response. The purpose of this study was to investigate whether patients with residual DCIS only have the same prognosis as those with no residual invasive or *in situ* disease. A retrospective analysis of a prospectively maintained database identified 435 patients, who received neoadjuvant chemotherapy for operable breast cancer between February 1985 and February 2003. Of these, 30 (7%; 95% CI 5–9%) had no residual invasive disease or DCIS and 20 (5%; CI 3–7%) had residual DCIS only. With a median follow-up of 61 months, there was no statistical difference in disease-free survival, 80% (95% CI 60–90%) in those with no residual invasive or *in situ* disease and 61% (95% CI 35–80%) in those with DCIS only (*P*=0.4). No significant difference in 5-year overall survival was observed, 93% (95% CI 75–98%) in those with no residual invasive or *in situ* disease and 82% (95% CI 52–94%) in those with DCIS only (*P*=0.3). Due to the small number of patients and limited number of events in each group, it is not possible to draw definitive conclusions from this study. Further analyses of other databases are required to confirm our finding of no difference in disease-free and overall survival between patients with residual DCIS and those with no invasive or *in situ* disease following neoadjuvant chemotherapy for breast cancer.

Neoadjuvant chemotherapy is now widely used in the treatment of locally advanced or potentially operable large breast cancers. Randomised trials have demonstrated neoadjuvant chemotherapy reduces the need for mastectomy (Powles *et al*, 1995; Fisher *et al*, 1998), with similar overall survival rates to adjuvant chemotherapy (Fisher *et al*, 1998). Women achieving no residual histological evidence of tumour after chemotherapy at the time of surgery (i.e. a pathological complete response (pCR)) have a significantly improved survival (Bonadonna et al, 1998; Fisher *et al*, 1998; Kuerer *et al*, 1999), and pCR is often used as an early surrogate marker of treatment efficacy. However, there is no standard method for grading pathological response of breast tumours to neoadjuvant chemotherapy and a number of different classification systems have been proposed (Chevallier *et al*, 1993; Sataloff *et al*, 1995; Fisher *et al*, 1997; Honkoop *et al*, 1998; Kuerer *et al*, 1998; Ogston *et al*, 2003). Most, but not all, of these grading schemes have included both no residual disease of any sort and residual ductal carcinoma *in situ* (DCIS) without invasive disease in the definition of pCR. We are unaware of published confirmatory data to justify this, and we have therefore reviewed the prognostic significance of residual DCIS alone following neoadjuvant chemotherapy compared with no histological evidence of any residual disease from our database. However, the number achieving residual DCIS only and no invasive or *in situ* disease is relatively small. For instance in NSABP B18, the largest randomised trial, 88 out 682 patients (13%) were classified as having no residual invasive disease following neoadjuvant chemotherapy (Wolmark *et al*, 2001). Similarly, in another large group of patients, treated within two prospective randomised trials, 43 of 372 patients (12%) were recorded as achieving a pCR (Kuerer *et al*, 1999). Thus, with a relatively small proportion of patients achieving a pCR following neoadjuvant chemotherapy, any comparison between those with residual DCIS and no invasive or *in situ* disease will inevitably involve small numbers of patients.

## PATIENTS AND METHODS

### Patient characteristics

A sequential and prospectively maintained database at the Royal Marsden Hospital, London, was retrospectively searched for women who had received neoadjuvant chemotherapy for primary operable breast cancer. Eligible patients had histologically confirmed invasive breast cancer on core biopsy prior to commencing chemotherapy and subsequently underwent surgery. Patients who received neoadjuvant therapy between 1985 and February 2003 were included in the study and data available until 31st March 2005 were used in the analysis. The presence of metastatic disease at diagnosis was excluded by chest radiograph, full blood count and standard serum biochemistry. Further investigations were performed if clinically indicated. Patients with locally advanced disease (defined as inoperable by the Haagensen criteria) or inflammatory breast cancer were excluded from this analysis (Haagensen and Stout, 1943).

### Pathological analysis

Although all patients were managed in the same institution, and hence followed departmental protocols, due to the time period, there was inevitably some difference in pathological assessment over time. The number of blocks examined to record residual disease varied with size of excision. Since the presence or absence of disease will vary depending on the amount of tissue examined, we looked at the mean and range of blocks taken in the group with no residual disease *vs* the group with residual *in situ* carcinoma only. The pathological data described in the study were assessed on the initial core biopsy. This has clear limitations, especially in assessing grade since mitotic counts over 10 high powered fields is not always possible on such small samples. Similarly, the oestrogen receptor (ER) staining can in rare cases be sufficiently heterogeneous to give misleading results in small core biopsy samples but in our experience, such profound heterogeneity leading to a change in status from positive to negative or *vice versa* is uncommon. We have included this data with the acknowledgement of this limitation. Involved surgical margins contained tumour at the margin or tumour transected at the margin. Close margins were defined as tumour within 1 mm of the margin.

### Treatment

Neoadjuvant chemotherapy regimens included: (1) anthracycline-based schedules comprising of doxorubicin 60 mg m^−2^ or epirubicin 60 mg m^−2^ once every 3 weeks, often within the context of clinical trials; (2) CMF (cyclophosphamide 100 mg orally days 1–14, methotrexate 30 mg m^−2^ days 1 and 8, and 5-fluorouracil 1 g m^−2^ days 1 and 8); and occasionally (3) mitoxantrone-containing regimens (up to 11 mg m^−2^). Treatment was usually given to a total of six courses, and occasionally to eight in specific trials.

Following each cycle of chemotherapy response was evaluated clinically by measurement of the two largest diameters of the tumour and graded according to the World Health Organisation (WHO) response criteria (Miller *et al*, 1981).

Breast conserving surgery or mastectomy was performed following chemotherapy. Conservative surgery involved macroscopic excision of the residual primary tumour with a surrounding margin of normal tissue. Patients with residual invasive carcinoma and microscopically involved radial margins (i.e. superior, inferior, medial or lateral) were offered either a mastectomy or wider excision. All patients who were treated with conservative surgery received adjuvant radiotherapy. The dose range of radiotherapy was 46–50 Gy, with boosts to the tumour bed ranging from 11.1 to 17.5 Gy. If the axilla was not surgically treated then radiotherapy was given to this region, with doses ranging from 46 to 50 Gy. If the axillary lymph nodes were involved then radiotherapy to the supraclavicular fossa was given, again dose ranging from 46 to 50 Gy. Radiotherapy was also given to patients with involved axillary lymph nodes after mastectomy. Adjuvant tamoxifen, 20 mg day^−1^, was administered to all patients, unless contraindicated. From 1997 onwards, patients who were ER and progesterone receptor (PgR) negative were not treated with tamoxifen.

### Follow-up

Clinical response was assessed after each cycle of chemotherapy. Following surgery, patients were reviewed every 3 months for 2 years and then 6 monthly until 5 years. Subsequently, yearly clinical and mammographic examinations were performed. Median follow-up was 5 years and 1 month.

### Statistical methods

Disease-free and overall survival were measured from the start of chemotherapy until relapse (death) or last follow-up. Survival curves were calculated by the method of Kaplan and Meier (1958) and differences were assessed by means of the log-rank statistic (Peto *et al*, 1977). Local recurrence free survival was measured from the start of chemotherapy until an isolated local recurrence and the data were censored in the event of a distant metastasis.

## RESULTS

Four hundred and thirty nine sequential patients treated with neoadjuvant treatment for operable breast cancer at the Royal Marsden Hospital between 1985 and February 2003 were considered for this analysis. Four were excluded because pathology was not available for review. Of the remaining 435 patients included in the review, 22% had a clinical complete response with a further 56% having a partial response (overall response rate 78%). At surgery 50 patients (11%) had either no residual disease of any sort (30; 7%, 95% CI 5–9%) or had residual ductal carcinoma *in situ* only (20; 5%, 95% CI 3–7%). The clinical and pathological characteristics of those with no residual invasive or *in situ* disease, residual DCIS and residual invasive carcinoma are given in Table 1. There were three patients in both the pCR and residual DCIS group who did not receive tamoxifen. The chemotherapy schedules administered are displayed in Table 2.

There were 385 (89%) patients with residual invasive carcinoma following neoadjuvant chemotherapy. Of these 385 patients, 90 had either minimal response, stable or progressive disease on clinical assessment following neoadjuvant treatment. Of those with residual carcinoma, 155 were treated with mastectomy and 230 with breast conserving surgery. This compared with 4 and 26 treated with mastectomy and breast conserving surgery respectively in the pCR group. Of all patients treated with mastectomy 102 patients received radiotherapy. For the residual DCIS group, eight were treated with mastectomy and 12 with breast conserving surgery. In the residual invasive carcinoma group, 41 patients underwent re-excision due to involved radial margins; 14 wider excision and 27 mastectomy. One patient in the residual DCIS group had a wider excision due to DCIS involving a radial margin. Of the 268 women treated with breast conservation as final surgery, invasive carcinoma involved a radial margin in 18 (7%) cases and was recorded as close in 11 (4%) patients. In 4 (2%) of the 268 patients DCIS involved a radial margin and in 18 (7%) the margin status was unknown.

For the group of patients with residual *in situ* disease, the mean number of slides was 18.8 (range 5–63). For the 30 patients in whom there was a complete response with no residual invasive or *in situ* disease, the mean number of slides examined was 17.4 (range 1–45). None of the patients had pathological axillary lymph node involvement. The extent of residual DCIS ranged from 1 to 80 mm, with four cases measuring over 50 mm. For seven patients, the exact measurement of residual DCIS was not available, and it was not possible to retrospectively ascertain the exact size.

**Figure 1:**
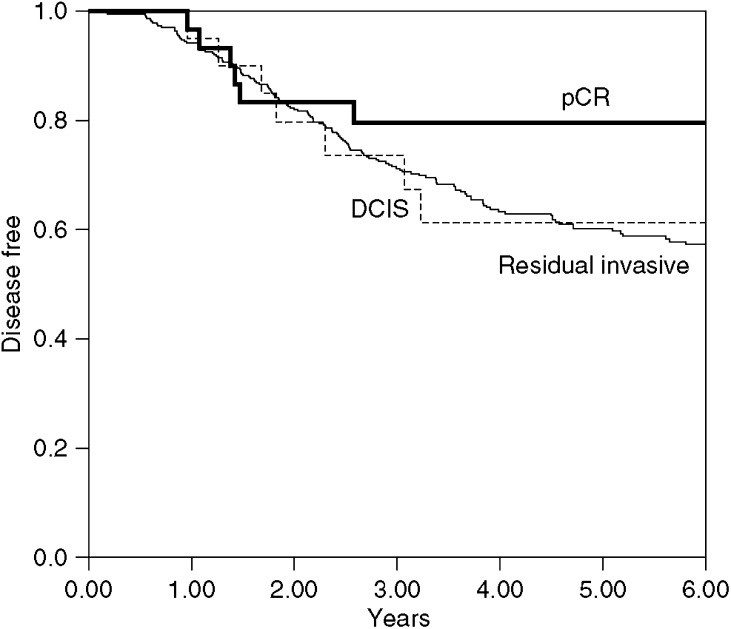
Disease-free survival in pathological complete response, residual DCIS and residual invasive carcinoma groups. There is no significant survival difference between pCR+DCIS *vs* residual invasive carcinoma group: *P*=0.07.

The 5-year disease-free survival in the pCR group was 80% (95% CI 60–90%) and in those with residual DCIS 61% (95% CI 35–80%) (*P*=0.4), see Figure 1. This compares with a 5-year disease-free survival of 60% (95% CI 55–65%) in those with residual invasive carcinoma. No significant difference in disease-free survival was observed between those classified as pathological complete responders (residual DCIS and no invasive or *in situ* disease) compared to those with residual invasive carcinoma, *P*=0.07.

**Figure 2:**
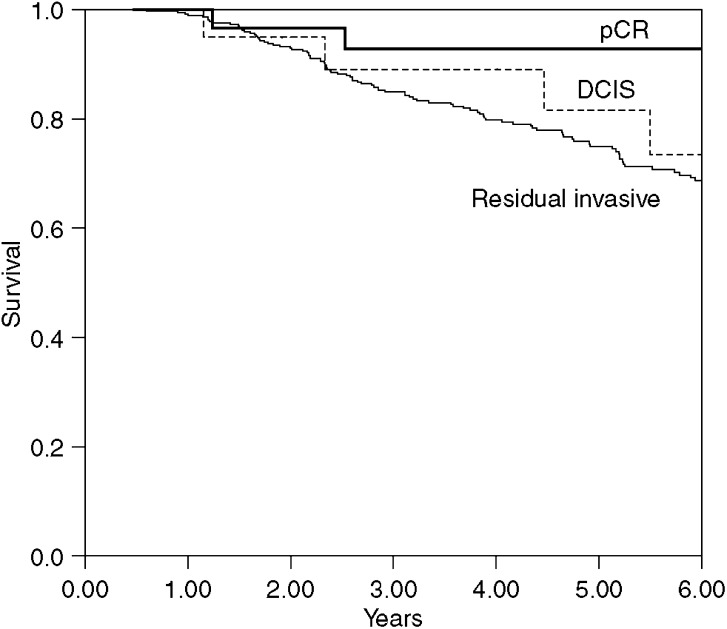
Overall survival in pathological complete response, residual DCIS and residual carcinoma groups. There is a significant survival difference between a combination of pCR and residual DCIS *vs* the residual invasive carcinoma group: *P*=0.04.

Five-year overall survival was 93% (95% CI 74–98%) in those with a pCR and 82% (95% CI 52–94%) in those with residual DCIS (*P*=0.3) (Figure 2). For the remaining 385 patients with residual invasive carcinoma, the 5-year overall survival was 75% (95% CI 70–79%). A significant difference in overall survival was observed between those with a pCR (i.e. residual DCIS and no invasive or *in situ* disease) and those with residual invasive carcinoma, *P*=0.04.

At the time of analysis, three patients in the pCR group had died, five in the residual DCIS group and 112 in the residual invasive carcinoma group.

The 5-year local recurrence free survival for those with pCR was 86% (95% CI 66–94%), which compared with 74% (95% CI 44–89%) in the residual DCIS group (*P*=0.5). In the residual invasive carcinoma group, the 5-year local recurrence free survival was 90% (95% CI 75–90%).

In 30 patients with pCR, there were 3 (10%) local recurrences and one (3%) contralateral nodal recurrence (axilla and supraclavicular fossa). Of these 30 patients, one also developed metastatic bone disease (3%). One woman (3%) developed high-grade DCIS in the ipsilateral breast.

In the group of 20 women with residual DCIS, there were 4 (20%) local recurrences and a further patient who developed a local recurrence simultaneously with pulmonary and pleural metastatic disease. One patient each recurred with brain and bone metastases.

Of the 385 patients with residual invasive carcinoma 32 (8%) had isolated local recurrences and a further 24 (6%) had concurrent local and distant recurrence.

## DISCUSSION

Few patients have no residual invasive and *in situ* disease or residual DCIS only following neoadjuvant chemotherapy for breast cancer. In our series, 30 (7%) achieved a pCR and 20 (5%) residual DCIS only. Of the patients with no residual disease, there were 3 (10%) local recurrences, 1 (3%) contralateral nodal recurrence and one patient developed-high grade DCIS in the ipsilateral breast. One patient in this group developed metastatic bone disease. In the residual DCIS group, there were 4 (20%) local recurrences and a further patient developing simultaneous local recurrence and metastatic disease. This compares with 32 (83%) isolated local recurrences and 24 (6%) concurrent local and distant recurrences in the residual invasive carcinoma group. With small numbers of patients and few events in each group this study is under powered to detect anything but a large difference in survival between the residual DCIS and pCR group. As a consequence, it is not possible to make definitive conclusions regarding the lack of difference in disease-free and overall survival between these two groups observed in this study. In addition, the very wide confidence intervals for both disease-free and overall survival in the two groups should be noted. Of the 268 patients treated with breast conserving surgery, 18 (7%) had radial margins involved by invasive carcinoma and 11 (4%) were recorded as having close surgical margins. These numbers compare favourably with another single institution series of 340 patients treated with neoadjuvant chemotherapy followed by breast conserving surgery (Chen *et al*, 2004).

There have been a number of proposed methods for assessing pathological response to neoadjuvant chemotherapy in the treatment of early and locally advanced breast cancer.

The Chevallier classification grades residual DCIS as a lesser response (class II) compared to complete disappearance of all tumour on microscopic analysis (class I) (Chevallier *et al*, 1993). Class III consists of invasive carcinomas with changes related to therapy and class IV have few or no changes following treatment. Chevallier and co-workers subsequently reported on 50 patients treated with an anthracycline-based neoadjuvant regimen (Chollet *et al*, 1997). In total, 11 (22%) cases were reported in class I and a further four (8%) in class II. Of these patients, 12 were in class III and 23 in class IV. However, overall and 5-year disease-free survival between those with residual *in situ* carcinoma and those with no residual invasive or *in situ* disease was not compared.

Recently, this group has investigated pCR in both breast and axillary lymph nodes following neoadjuvant chemotherapy (Chollet *et al*, 2002). Retrospectively, they analysed 451 patients, of whom only 171 did not have clinically involved lymphadenopathy. In total, 10 (13.2%) women had a class I response and 5 (6.6%) were reported as class II. There were 41 (53.9%) and 20 (26.3) patients in class 3 and 4, respectively. Patients in classes I and II according to the Chevallier system had an improved 15-year disease-free survival compared with those in classes III and IV (*P*=0.0053). However, when class I was considered alone against the other classes, there was still a significant difference in disease-free survival. Overall survival was not significantly different between those with residual invasive disease and those in Chevalier class I+II. However, a significant difference in overall survival (*P*=0.047) between class I tumours and those with residual disease was observed.

Other classification systems have included those with no evidence of residual *in situ* or invasive carcinoma together with those tumours that have residual *in situ* disease.

In the NSABP B-18 trial, histological response was divided into two groups, those with a pCR (including residual *in situ* disease) and those with residual invasive carcinoma (Fisher *et al*, 1997). This trial and the accompanying classification system are important due to the number of patients recruited; 1523 women were randomised between preoperative or postoperative AC (doxorubicin and cyclophosphamide) chemotherapy. There was no difference in disease-free survival or distant disease-free survival between the two groups, but more patients who received neoadjuvant chemotherapy compared to those given adjuvant therapy underwent lumpectomy and radiotherapy (67.8 vs 59.8% respectively). Relapse-free survival was significantly better in those who achieved a pCR with neoadjuvant treatment compared to those with residual invasive carcinoma.

Another publication with a large number of patients treated in two prospective randomised trials utilised the classification system proposed by Kuerer *et al* (1999). This has three grades; no evidence of residual disease, <1 cm^3^ of macroscopic residual tumour (residual microscopic foci of cancer cells included) and >1 cm^3^ of residual macroscopic tumour (Kuerer *et al*, 1998). These investigators analysed 372 patients with locally advanced breast cancer treated with four cycles of neoadjuvant chemotherapy in two prospective randomised studies (Kuerer *et al*, 1999). Of these patients, 43 (12%) had no histological evidence of residual invasive carcinoma in both breast and axillary nodes. The patients included in the pCR group had a significantly better 5-year overall and disease-free survival than in those with less than pathologic complete response.

Recently, Miller and Payne have developed a novel 5 grade system based on the degree of tumour cell loss secondary to neoadjuvant chemotherapy (Ogston *et al*, 2003). Grade 5 in this classification is defined by the presence of no malignant cells, only vascular fibroelastic stroma, but the presence of DCIS is permitted. A loss of more than 90% of tumour cells is classified as grade 4. An estimated reduction between 30 and 90% of tumour cells is graded as 3 and those with up to 30% loss of tumour cells as grade 2. No change or some alteration to individual malignant cells but reduction in cellularity is classified as grade 1. A consecutive series of 176 patients with large and locally advanced breast cancers were studied. In total, 170 of these cases were available for analysis. In total, 26 patients (15%) were included in grade 1, 41 (24%) had a grade 2 response and 46 (27%) were classified as grade 3. A total of 34 women (20%) had a grade 4 response and 23 (14%) were classified as grade 5. There was a statistically significant difference in terms of disease-free and overall survival between those classified as grade 5 with all other patients. On multivariate analysis, increasing pathological grade of response, negative oestrogen receptor status and absence of histologically detectable residual disease in axillary nodes following chemotherapy were independent predictors of improved overall survival in this group of patients.

A further system by Honkoop describes two grades – gross residual or minimal residual disease, the latter including no residual invasive cancer or scattered foci of cancer (Honkoop *et al*, 1998). A total of 42 women treated with neoadjuvant chemotherapy followed by mastectomy and radiotherapy were studied. On multivariate analysis, an improved disease-free and overall survival were observed in those with a better pathological response.

Sataloff *et al* (1995) proposed a system, which consists of four pathological grades: complete response including DCIS, greater than 50% therapeutic effect but less than total or near total, less than 50% therapeutic effect but treatment change evident and no effect. They performed a retrospective review of 36 patients with locally advanced breast cancer treated with three cycles of neoadjuvant chemotherapy. A modified radical mastectomy was performed in all cases and adjuvant chemotherapy (consisting of the same agents) was administered followed by chest wall radiotherapy. On pathological examination of the surgical specimen a complete response, including DCIS, was observed in 14 cases (39%). Five tumours (14%) were reported as having greater than 50% therapeutic effect but less than total. A less than 50% therapeutic effect was seen in 12 (33%) of the 36 patients. No pathologic response was demonstrated in 5 (14%) tumours. In this series, axillary lymph node involvement was observed in 22 (61%) of cases. Recurrences occurred in 20 (56%) of patients and these all presented with distant metastases. A statistically significant difference in survival between patients in the complete pathological response and the other pathological groups was demonstrated.

With most classification systems of grading pathological response post neoadjuvant chemotherapy including patients with residual DCIS in the pCR category, it is important to ascertain whether there is a difference in prognosis between those with no invasive or *in situ* disease and those with residual DCIS.

Even in the two large trials discussed, the number achieving residual DCIS only or no invasive or *in situ* disease is relatively small. In the NSABP B18 trial, 88 out 682 patients (13%) were classified as having no residual invasive disease (Wolmark *et al*, 2001) and similarly in the 372 patients reported by Kuerer *et al* (1999), only 43 (12%) achieved a pCR. Thus, with a relatively small proportion of patients achieving a pCR following neoadjuvant chemotherapy, any comparison between those with residual DCIS and no invasive or *in situ* disease will inevitably involve relatively small numbers of patients. As a consequence, our data are based on a small number of cases, but they represent so far the only results of which we are aware supporting the practice of including patients with residual DCIS in the group of pathological complete responders following neoadjuvant chemotherapy. Although we have not been able to demonstrate a disease-free or overall survival advantage for women with no residual invasive or *in situ* disease compared to those with residual DCIS, our numbers are small with relatively few events in each group and thus definitive conclusions cannot be made. This study is under powered to detect anything but a large difference in survival, and thus further analysis of large prospective randomised trials and other databases are required to confirm these findings.

## Figures and Tables

**Table 1:** Patient characteristics

**Patient characteristic**	**pCR**	**Residual DCIS**	**Residual invasive**
*Age (years)*			
Median (range)	48 (31–62)	43 (26–54)	48 (24–67)
			
*Menopausal status*			
Pre	17	17	220
Peri	4	0	35
Post	7	2	100
Hysterectomy	2	1	30
			
*ER status*			
Positive	11	10	250
Negative	16	7	88
Not known	3	3	47
			
*Chemotherapy*			
Anthracycline	29	19	311
Nonanthracycline	1	1	74
			
*Surgery*			
Conservative	26	12	230
Mastectomy	4	8	155

**Table 2:** Chemotherapy regimens administered to pathological complete response and residual DCIS groups

**Chemotherapy**	**Number DCIS group**	**Number pCR group**
ECisF	6	8
AC	10	17
NE	2	3
FEC	1	1
NM	0	1
CMF–AC	1	0

ECisF: Epirubicin 60 mg m^−2^, day 1 of 3 week cycle; Cisplatin 60 mg m^−2^, day 1 of 3 week cycle; 5 Fluorouracil 200 mg m^−2^, 24 h continuous infusion for 21 weeks.

AC: Doxorubicin 60 mg m^−2^, day 1 of 3 week cycle; Cyclophosphamide 600 mg m^−2^, day 1 of 3 week cycle.

NE: Navelbine 25 mg m^−2^, day 1+8 of 3 week cycle (maximum dose 60 mg); Epirubicin 60 mg m^−2^, day 1 of 3 week cycle.

FEC: 5 Fluorouracil 600 mg m^−2^, day 1 of 3 week cycle; Epirubicin 60 mg m^−2^, day 1 of 3 week cycle; Cyclophosphamide 600 mg m^−2^, day 1 of 3 week cycle.

NM: Navelbine 25 mg m^−2^, day 1+8 of 3 week cycle; Mitoxantrone 12 mg m^−2^, day 1 of 3 week cycle.

CMF: Cyclophosphamide 100 mg m^−2^, once daily orally days 1–14; Methotrexate 40 mg m^−2^, day 1+8 of 4 week cycle; 5 Fluorouracil 600 mg m^−2^, day 1+8 of 4 week cycle.
